# Skeletal Muscle Damage in COVID-19: A Call for Action

**DOI:** 10.3390/medicina57040372

**Published:** 2021-04-12

**Authors:** Amira Mohammed Ali, Hiroshi Kunugi

**Affiliations:** 1 Department of Psychiatric Nursing and Mental Health, Faculty of Nursing, Alexandria University, Alexandria 21527, Egypt; 2 Department of Behavioral Medicine, National Institute of Mental Health, National Center of Neurology and Psychiatry, Tokyo 187-8553, Japan; 3 Department of Psychiatry, School of Medicine, Teikyo University, Tokyo 173-8605, Japan; hkunugi@ncnp.go.jp; 4 Department of Mental Disorder Research, National Institute of Neuroscience, National Center of Neurology and Psychiatry, Tokyo 187-8551, Japan

**Keywords:** coronavirus disease 2019, COVID-19, cytokine storm, intensive care unit-acquired weakness, older adults, aging, skeletal muscle, musculoskeletal, myoglobin, rhabdomyolysis, malnutrition, severity

## Abstract

Both laboratory investigations and body composition quantification measures (e.g., computed tomography, CT) portray muscle loss in symptomatic Coronavirus disease 2019 (COVID-19) patients. Muscle loss is associated with a poor prognosis of the disease. The exact mechanism of muscle damage in COVID-19 patients, as well as the long-term consequences of muscle injury in disease survivors, are unclear. The current review briefly summarizes the literature for mechanisms, assessment measures, and interventions relevant to skeletal muscle insult in COVID-19 patients. Muscle injury is likely to be attributed to the cytokine storm, disease severity, malnutrition, prolonged physical inactivity during intensive care unit (ICU) stays, mechanical ventilation, and myotoxic drugs (e.g., dexamethasone). It has been assessed by imaging and non-imaging techniques (e.g., CT and electromyography), physical performance tests (e.g., six-minute walk test), anthropometric measures (e.g., calf circumference), and biomarkers of muscle dystrophy (e.g., creatine kinase). Interventions directed toward minimizing muscle loss among COVID-19 patients are lacking. However, limited evidence shows that respiratory rehabilitation improves respiratory function, muscle strength, quality of life, and anxiety symptoms in recovering older COVID-19 patients. Neuromuscular electrical stimulation may restore muscle condition in ICU-admitted patients, albeit empirical evidence is needed. Given the contribution of malnutrition to disease severity and muscle damage, providing proper nutritional management for emaciated patients may be one of the key issues to achieve a better prognosis and prevent the after-effects of the disease. Considerable attention to longer-term consequences of muscle injury in recovering COVID-19 patients is necessary.

## 1. Overview

Coronavirus disease 2019 (COVID-19) is a viral infection that develops following the access of severe acute respiratory syndrome-coronavirus-2 (SARS-CoV-2) to the respiratory tract. SARS-CoV-2 sometimes induces severe inflammatory and oxidative stress, which injure pulmonary alveoli resulting in the development of severe acute respiratory distress syndrome (ARDS), bilateral viral pneumonia, and respiratory failure [1,2,3]. Moreover, SARS-CoV-2 particles have been isolated from different body tissues, including the intestine, the central nervous system, and cardiac muscle. COVID-19 mortality is higher among people with cardiac diseases, denoting that it may directly impede cellular processes in tissues outside the respiratory tract [4,5].

Skeletal muscle, the largest body tissue involved in glucose metabolism [6,7], seems to be among tissues affected by SARS-CoV-2 [8,9]. Muscle pain is one of the key symptoms that develop during the first three days of infection in people who get hospitalized due to contracting SARS-CoV-2 [10,11,12]. Meta-analytic studies indicate that myalgia (muscle soreness)/fatigue is the third most common symptom (after unremitting fever and cough) in people with symptomatic SARS-CoV-2 infection [8,9]. The duration of myalgia depends primarily on disease severity [12,13]. Imaging data from hospitalized patients in China showed that muscle ache on admission is associated with abnormal images of the lung, and it predicts poor prognosis, especially in older people [14]. The exact mechanism of muscle damage in COVID-19 patients, as well as the long-term consequences of muscle injury in disease survivors, are unclear. In an attempt to bridge the gap, the current article explored the literature for these three aspects.

## 2. Possible Mechanisms of Muscle Damage in COVID-19

**Figure 1:**
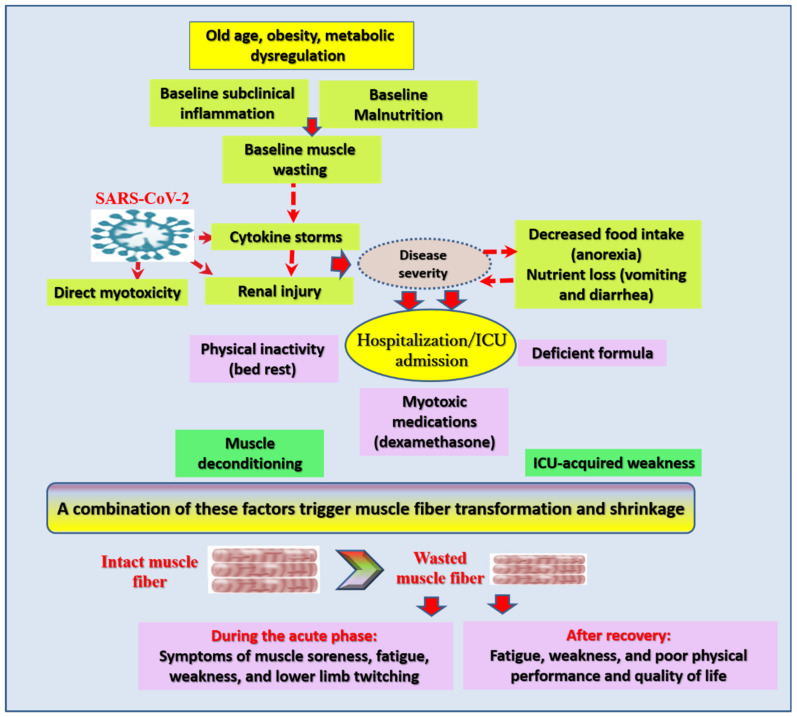
Possible factors contributing to skeletal muscle injury in Coronavirus disease 2019 (COVID-19) patients. Abbreviations: SARS-CoV-2: severe acute respiratory syndrome-coronavirus-2, ICU: intensive care unit. Catabolic molecules released in individuals with baseline sarcopenia and obesity usually occurring in older adults and comorbid conditions, secondary to inflammaging and malnutrition, along with the cytokine storms induced by SARS-CoV-2, may directly injure muscular structures. Energy imbalance associated with gastrointestinal symptoms, muscle deconditioning by prolonged bed rest, myotoxic drugs such as dexamethasone, deficient nutrition, the cytokine storm, and mechanical ventilation contribute to ICU-acquired weakness among critical COVID-19 patients.

Excessive production of proinflammatory cytokines in hypercatabolic conditions is associated with oxidative stress, which promotes the production of corrosive molecules that cause severe myocyte damage [15]. The exact dynamics underlying muscle wasting in COVID-19 are unclear. However, researchers suggest that muscle loss in COVID-19 patients is an outcome of a wide range of interrelated factors (Figure 1). Old age, as well as metabolic and inflammatory disorders (diabetes, obesity, cardiovascular diseases, cancer, etc.), are associated with baseline states of protein-energy malnutrition and systemic inflammation/inflammaging. Apart from COVID-19, these individuals usually express some degree of muscle wasting (sarcopenia); they are also a majority of COVID-19 patients. In fact, older adults affected by SARS-CoV-2 are likely to express dramatic injuries in muscular structure, especially at the late stages of the disease [16,17]. Myokines and adipokines produced by sarcopenic muscle and adipose tissue stimulate signaling of inflammation and oxidative stress resulting in hyper-catabolism, especially in people with advanced age and metabolic disorders [18,19]. Malnutrition and baseline inflammation are thought to be key effectors in the development of cytokine storms in these individuals when they contract COVID-19 [3]. Excessive cytokines directly induce multiple organ damage, including skeletal muscle, which undergoes degenerative transformation and shrinkage [15]. They also interfere with hormones that promote muscular integrity, such as testosterone [19]. In rare cases, viral-induced myotoxicity was reported [13]. Renal injury noted by macrohematuria or even renal failure is reported along with myalgia in COVID-19 patients with rhabdomyolysis, denoting a possible renal involvement in COVID-19-related muscle damage [13,20]. Symptoms conducive to malnutrition, such as anorexia, nausea, and vomiting, are common in some COVID-19 patients pinpointing insufficient energy supply—a primary trigger for the breakdown of body protein to fuel metabolic needs [21,22,23,24,25,26].

Generalized wasting was reported in COVID-19 patients treated at home; however, greater weight loss was evident in hospital and intensive care unit (ICU)-admitted patients, with more extensive muscle loss in obese patients [27,28]. Although hospital/ICU admitted COVID-19 patients are expected to be more severe than patients treated at home, several other factors may promote muscle breakdown in ICU admitted COVID-19 patients: (1) bed rest and being in the prone position for a long time is associated with muscle deconditioning [29,30,31], (2) certain antiviral drugs (e.g., hydroxychloroquine) and medications prescribed to critical patients, such as dexamethasone, evoke muscle damage, with a greater insult in immobile patients [13,15], (3) undernutrition associated with deficient or inappropriate nutritional formula may trigger muscle breakdown for the provision of necessary energy, (4) ICU-acquired weakness develops in critical patients with systemic inflammation (higher blood levels of interleukin (IL)-6, IL-8, IL-10, and fractalkine) [32]—severe COVID-19 patients are susceptible to this condition as one of the disease complications because of the cytokine storm, and (5) intubation and mechanical ventilation trigger body protein loss, including muscle protein, over a short period of time. Patients with a prolonged hospital stay, severe course of the disease, and cytokine storm express a considerable need to be followed up within 100 days following discharge from the hospital [29,30,31,33].

## 3. Available Measures of Muscular Assessment in COVID-19 Patients

Weight loss is common in critical and recovering COVID-19 patients, and it usually affects non-fat mass, especially in obese patients [3,27,28,29]. Several lines of evidence report dystrophic damages of skeletal muscle in COVID-19 patients [16,17,29,34,35,36]. Quantification of lean body mass (LBM) in COVID-19 patients one and 20 days after admission to the ICU through computed tomography (CT) revealed a significant LBM loss, along with serious metabolic alterations, especially in obese patients [29]. CT is one of the gold standard techniques that accurately measure body composition and quantify muscle mass [29,37]. Pectoralis muscle mass was measured by chest CT, primarily conducted to examine the extent of lung fibrosis. Pectoralis muscle index (cross-sectional areas of the pectoralis muscle/patient’s height square (m^2^)) is a valid frailty index that can predict pulmonary functioning, length of hospital stays, and survival [37]. Magnetic resonance imaging (MRI) identified myositis in a case of COVID-19-related rhabdomyolysis [13]. It also uncovered a reduction in the maximal voluntary contraction for quadriceps and biceps in recovering patients (54% and 69% of the predicted normal value, respectively) [38]. Skeletal muscle strength and physical performance have been evaluated 2–3 months after hospital discharge in remitting patients through spirometry, six-minute walk test, physical performance (1-min sit-to-stand and short physical performance battery tests), and cardiopulmonary exercise test [38,39]. Assessment of muscle loss indicated by calf circumference along with body mass index (BMI) may be integrated with other clinical indicators, such as hypoalbuminemia and having a diagnosis of diabetes mellitus, to predict nutritional risk and the progression to severe disease status in hospitalized patients with COVID-19 [40]. It is worth mentioning that body size and hydration (obesity and electrolyte imbalance are common in severe COVID-19 [3]) may confound measures commonly used for muscle mass evaluation, such as dual-energy X-ray absorptiometry, total and partial body potassium, and bio-electrical impedance analysis. Therefore, gold standard measures (e.g., CT and MRI) may be preferred for obese and edematous patients [41,42].

Laboratory reports showed remarkable elevation in biomarkers of muscle loss, such as creatine kinase (CK), in up to 27% of hospitalized COVID-19 patients, a condition described as hyperCKemia [16,36]. The incidence of hyperCKemia in severe COVID-19 is higher in older males who demonstrate comorbidities. Analysis adjusted for demographic characteristics and disease severity showed that higher CK levels are associated with the rise in inflammation markers [16]. Several case studies report the development of rhabdomyolysis in COVID-19 patients encountering ARDS and renal insufficiency [17,34,35,43]. Some of these patients were relatively young—aged 35 to 49 years [34,35]. Rhabdomyolysis describes a condition of severe muscle injury, which manifests by intense muscle soreness, fatigue, weakness, and lower limb pain/twitching, along with alterations in muscular biomarkers, such as CK, lactate dehydrogenase (LDH), and myoglobin—a heme-containing globular protein abundant in myocytes [17]. Meta-analytic studies report elevated levels of CK [44,45,46], LDH [46], and myoglobin [44,45,46] in severe COVID-19 patients than in mild conditions. Some of these studies associate high myoglobin and CK with cardiac muscle injury [44,45]. All these reports support the possibility that muscle injury and subsequent rhabdomyolysis, which are often severe in older people, represent a characteristic feature of SARS-CoV-2 infection [17,34,35].

## 4. Long Term Consequences of Muscle Injury in COVID-19 Patients

Longitudinal reports from previous SARS pandemics (e.g., SARS-2002 and MERS) showed that compared with healthy age- and gender-matched adults, survivors of moderate and severe SARS infections express cardiorespiratory and musculoskeletal dysfunctions [47]. Of interest, participants who were hospitalized for three weeks manifested 32% and 13% reductions in grip strength and 6-minute distance walk tests, respectively, 2 to 3 months after discharge from the hospital, which was associated with low scores on the physical domain of the 36-Item Short-Form Health Survey (SF-36) indicating poor health-related quality of life [47]. Although the available reports on muscle insult in COVID-19 patients stem from cross-sectional studies, follow up studies report that muscular deficits in COVID-19 patients display apparent similarities with those noted in the previous pandemics caused by coronaviruses: reduced muscular strength was associated with dyspnea and fatigue while performing activities of daily living 2–3 months after discharge. Poor quality of life and depression were common among those patients [36,38,39]. A patient recovering from rhabdomyolysis could walk normally after 43 days, but he manifested reduced endurance [43]. In that patient, motor and sensory fiber injuries (axonal injury accompanied by demyelination) in both legs were detected by electromyography after 120 days [43]. In another study, electromyography revealed a decrease in compound muscle action potential of the motor nerves despite normal nerve conduction in a 58-years old male COVID-19 patient with rhabdomyolysis [20]. Such reports highlight the possibility that COVID-19-related muscular injury may cause long-term disabilities.

## 5. Options for Minimizing, Preventing, and Treating Muscle Damage in COVID-19 Patients

Age-related muscle loss—similar to muscle loss in COVID-19 though the former is gradual in nature—is commonly reversed by high protein supplementation [48,49]. Metanalytic studies show increased muscle strength in frail older adults receiving a combined supplement of proteins and vitamin D (100–1600 IU/day) with no effect on muscle mass or physical performance [48], whereas when protein supplementation was combined with muscle-strengthening exercise, it resulted in improved whole LBM, appendicular mass, leg strength, and walking capability [49]. Malnutrition is a possible cause of immunological and muscular dysfunction in COVID-19 [3,15,19]. Hence, muscle damage in vulnerable COVID-19 patients (vulnerability indicated by changes in body weight, muscle mass, muscle strength, anthropometry, and biomarkers) may be minimized by suitable nutritional support: oral intake of protein-rich food/dietary supplements or volume-controlled higher-protein enteral formula according to patients’ status [50,51]. In fact, molecular modeling studies show that marine proteins hydrolyzed by gastrointestinal enzymes generate active peptides that can effectively interact with SARS-CoV-2 main protease and monoamine oxidase A, which can probably limit viral load and associated severity [52]. In support of such reports, clinical evidence shows that COVID-19 patients receiving L-Glutamine exhibited shorter hospital stay, fewer ICU admissions, and lower mortality than non-supplemented patients [53]. Therefore, the effect of protein and amino acid supplementation on myopathy in COVID-19 may also be secondary to decreased disease severity and immobility that are associated with a prolonged ICU stay, which predisposes to ICU-acquired weakness, albeit further investigations are necessary to test this hypothesis. Whenever possible, exposure to sunlight should be encouraged to stimulate vitamin D synthesis, which promotes immunity and muscle protein synthesis [2,19].

Neuromuscular electrical stimulation—inducing muscle contraction by applying small electrical impulses—is suggested to be used to maintain muscular blood flow, reduce muscle atrophy, and improve muscle strength in ICU-admitted COVID-19 patients [54]. A quasi-experimental study implementing a six-week program of respiratory rehabilitation among recovering older adults with COVID-19 reported significantly improved pulmonary function, six-minute walk functional test, quality of life (all eight dimensions of the SF-36), and anxiety, with no effect on depression [55]. Home-based exercise programs are reported to promote physical fitness as well as muscle mass and strength in healthy community-dwelling older adults [56]. Resistance exercise is also recommended to achieve these goals in recovering COVID-19 patients [15,19].

## 6. Conclusions

In conclusion, this work presents evidence of muscular damage in COVID-19 patients, especially hospitalized and ICU-admitted, which raises the question of whether ICU-acquired weakness is a complication of COVID-19. It also emphasizes the importance of assessing muscle condition, particularly in older, comorbid, and severe patients, using gold-standard measures (e.g., CT and MRI). It is important to evaluate the muscle-saving potential of techniques known to maintain muscular integrity (such as neuromuscular electrical stimulation) in ICU-admitted patients through rigorously designed clinical trials. Providing proper nutritional support (e.g., protein and amino acid supplements) along with an adequate level of physical activity/electrical stimulation may be necessary to restore skeletal muscle metabolism and structure in the victims of COVID-19 and prevent the after-effects of physical disability in recovering patients. Enough exposure to sunlight, both in recovering patients as well as in COVID-19 vulnerable groups, is important for vitamin D synthesis and related immunity and muscular-related benefits.

## Data Availability

Not applicable.

## Abbreviations

ARDS	Acute respiratory distress syndrome
CK	Creatine kinase
COVID-19	Coronavirus disease 2019
CT	Computed tomography
ICU	Intensive care unit
IL	Interleukin
LDH	Lactate dehydrogenase
MRI	Magnetic resonance imaging
SARS-CoV-2	Severe acute respiratory syndrome-coronavirus-2
SF-36	36-Item Short-Form Health Survey

## References

[1] Zhou Y., Fu B., Zheng X., Wang D., Zhao C., Qi Y., Sun R., Tian Z., Xu X., Wei H. (2020). Pathogenic T-cells and inflammatory monocytes incite inflammatory storms in severe COVID-19 patients. Natl. Sci. Rev..

[2] Xu Y., Baylink D.J., Chen C.S., Reeves M.E., Xiao J., Lacy C., Lau E., Cao H. (2020). The importance of vitamin d metabolism as a potential prophylactic, immunoregulatory and neuroprotective treatment for COVID-19. J. Transl. Med..

[3] Ali A.M., Kunugi H. (2021). Approaches to nutritional screening in patients with Coronavirus Disease 2019 (COVID-19). Int. J. Environ. Res. Public Health.

[4] Wang W., Xu Y., Gao R., Lu R., Han K., Wu G., Tan W. (2020). Detection of SARS-CoV-2 in Different Types of Clinical Specimens. JAMA.

[5] Tavazzi G., Pellegrini C., Maurelli M., Belliato M., Sciutti F., Bottazzi A., Sepe P.A., Resasco T., Camporotondo R., Bruno R. (2020). Myocardial localization of coronavirus in COVID-19 cardiogenic shock. Eur. J. Heart Fail..

[6] Riuzzi F., Sorci G., Sagheddu R., Chiappalupi S., Salvadori L., Donato R. (2018). RAGE in the pathophysiology of skeletal muscle. J. Cachexia Sarcopenia Muscle.

[7] Egawa T., Ohno Y., Yokoyama S., Yokokawa T., Tsuda S., Goto K., Hayashi T. (2019). The Protective Effect of Brazilian Propolis against Glycation Stress in Mouse Skeletal Muscle. Foods.

[8] Nasiri M.J., Haddadi S., Tahvildari A., Farsi Y., Arbabi M., Hasanzadeh S., Jamshidi P., Murthi M., Mirsaeidi M. (2020). COVID-19 clinical characteristics, and sex-specific risk of mortality: Systematic Review and Meta-analysis. Front. Med..

[9] Zhu J., Ji P., Pang J., Zhong Z., Li H., He C., Zhang J., Zhao C. (2020). Clinical characteristics of 3062 COVID-19 patients: A meta-analysis. J. Med. Virol..

[10] Vacchiano V., Riguzzi P., Volpi L., Tappatà M., Avoni P., Rizzo G., Guerra L., Zaccaroni S., Cortelli P., Michelucci R. (2020). Early neurological manifestations of hospitalized COVID-19 patients. Neurol. Sci..

[11] Nidadavolu L., Walston J. (2020). Underlying Vulnerabilities to the Cytokine Storm and Adverse COVID-19 Outcomes in the Aging Immune System. J. Gerontol. A Biol. Sci. Med. Sci..

[12] Paliwal V.K., Garg R.K., Gupta A., Tejan N. (2020). Neuromuscular presentations in patients with COVID-19. Neurol. Sci..

[13] Finsterer J., Scorza F. (2021). SARS-CoV-2 associated rhabdomyolysis in 32 patients. Turk. J. Med. Sci..

[14] Zhang X., Cai H., Hu J., Lian J., Gu J., Zhang S., Ye C., Lu Y., Jin C., Yu G. (2020). Epidemiological, clinical characteristics of cases of SARS-CoV-2 infection with abnormal imaging findings. Int. J. Infect. Dis..

[15] Welch C., Greig C., Masud T., Wilson D., Jackson T.A. (2020). COVID-19 and Acute Sarcopenia. Aging Dis..

[16] Pitscheider L., Karolyi M., Burkert F.R., Helbok R., Wanschitz J.V., Horlings C., Pawelka E., Omid S., Traugott M., Seitz T. (2020). Muscle involvement in SARS-CoV-2 infection. Eur. J. Neurol..

[17] Jin M., Tong Q. (2020). Rhabdomyolysis as Potential Late Complication Associated with COVID-19. Emerg. Infect. Dis..

[18] Breucker S.D., Luce S., Njemini R., Bautmans I., Decoster L., Mets T., Pepersack T. (2020). Analysis of inflammatory markers and hormones in old cancer patients: A descriptive study. Exp. Gerontol..

[19] Zhou L., Liu C., Yang C. (2021). Comment on ‘COVID-19: A major cause of cachexia and sarcopenia’ by Morley et al. J. Cachexia Sarcopenia Muscle.

[20] Rosato C., Bolondi G., Russo E., Oliva A., Scognamiglio G., Mambelli E., Longoni M., Rossi G., Agnoletti V. (2020). Clinical, electromyographical, histopathological characteristics of COVID-19 related rhabdomyolysis. SAGE Open Med. Case Rep..

[21] Zhan T., Liu M., Tang Y., Han Z., Cheng X., Deng J., Chen X., Tian X., Huang X. (2020). Retrospective analysis of clinical characteristics of 405 patients with COVID-19. J. Int. Med. Res..

[22] Cheng A., Hu L., Wang Y., Huang L., Zhao L., Zhang C., Liu X., Xu R., Liu F., Li J. (2020). Diagnostic performance of initial blood urea nitrogen combined with D-dimer levels for predicting in-hospital mortality in COVID-19 patients. Int. J. Antimicrob. Agents.

[23] Liu G., Zhang S., Mao Z., Wang W., Hu H. (2020). Clinical significance of nutritional risk screening for older adult patients with COVID-19. Eur. J. Clin. Nutr..

[24] Garrigues E., Janvier P., Kherabi Y., Le Bot A., Hamon A., Gouze H., Doucet L., Berkani S., Oliosi E., Mallart E. (2020). Post-discharge persistent symptoms and health-related quality of life after hospitalization for COVID-19. J. Infect..

[25] Zhong H., Wang Y., Zhang Z.L., Liu Y.X., Le K.J., Cui M., Yu Y.T., Gu Z.C., Gao Y., Lin H.W. (2020). Efficacy and safety of current therapeutic options for COVID-19—Lessons to be learnt from SARS and MERS epidemic: A systematic review and meta-analysis. Pharmacol. Res..

[26] Du X., Liu Y., Chen J., Peng L., Jin Y., Cheng Z., Wang H.H.X., Luo M., Chen L., Zhao Y. (2020). Comparison of the Clinical Implications among Two Different Nutritional Indices in Hospitalized Patients with COVID-19. medRxiv.

[27] Haraj N.E., El Aziz S., Chadli A., Dafir A., Mjabber A., Aissaoui O., Barrou L., El Kettani El Hamidi C., Nsiri A., Al Harrar R. (2021). Nutritional status assessment in patients with Covid-19 after discharge from the intensive care unit. Clin. Nutr. ESPEN.

[28] Di Filippo L., De Lorenzo R., D’Amico M., Sofia V., Roveri L., Mele R., Saibene A., Rovere-Querini P., Conte C. (2020). COVID-19 is associated with clinically significant weight loss and risk of malnutrition, independent of hospitalisation: A post-hoc analysis of a prospective cohort study. Clin. Nutr..

[29] Gualtieri P., Falcone C., Romano L., Macheda S., Correale P., Arciello P., Polimeni N., Lorenzo A. (2020). Body Composition Findings by Computed Tomography in SARS-CoV-2 Patients: Increased Risk of Muscle Wasting in Obesity. Int. J. Mol. Sci..

[30] Zeppa S.D., Agostini D., Piccoli G., Stocchi V., Sestili P. (2020). Gut Microbiota Status in COVID-19: An Unrecognized Player?. Front. Cell Infect. Microbiol..

[31] Zhao X., Li Y., Ge Y., Shi Y., Lv P., Zhang J., Fu G., Zhou Y., Jiang K., Lin N. (2020). Evaluation of Nutrition Risk and Its Association with Mortality Risk in Severely and Critically Ill COVID-19 Patients. J. Parenter. Enter. Nutr..

[32] Witteveen E., Wieske L., van der Poll T., van der Schaaf M., van Schaik I.N., Schultz M.J., Verhamme C., Horn J. (2017). Increased Early Systemic Inflammation in ICU-Acquired Weakness; A Prospective Observational Cohort Study. Crit. Care Med..

[33] De Lorenzo R., Conte C., Lanzani C., Benedetti F., Roveri L., Mazza M.G., Brioni E., Giacalone G., Canti V., Sofia V. (2020). Residual clinical damage after COVID-19: A retrospective and prospective observational cohort study. PLoS ONE.

[34] Alrubaye R., Choudhury H. (2020). Severe Rhabdomyolysis in a 35-Year-old Woman with COVID-19 due to SARS-CoV-2 Infection: A Case Report. Am. J. Case Rep..

[35] Mukherjee A., Ghosh R., Aftab G. (2020). Rhabdomyolysis in a Patient with Coronavirus Disease 2019. Cureus.

[36] Disser N.P., De Micheli A.J., Schonk M.M., Konnaris M.A., Piacentini A.N., Edon D.L., Toresdahl B.G., Rodeo S.A., Casey E.K., Mendias C.L. (2020). Musculoskeletal Consequences of COVID-19. J. Bone Jt. Surg. Am..

[37] Ufuk F., Demirci M., Sagtas E., Akbudak I.H., Ugurlu E., Sari T. (2020). The prognostic value of pneumonia severity score and pectoralis muscle Area on chest CT in adult COVID-19 patients. Eur. J. Radiol..

[38] Paneroni M., Simonelli C., Saleri M., Bertacchini L., Venturelli M., Troosters T., Ambrosino N., Vitacca M. (2021). Muscle Strength and Physical Performance in Patients Without Previous Disabilities Recovering From COVID-19 Pneumonia. Am. J. Phys. Med. Rehabil..

[39] Raman B., Cassar M.P., Tunnicliffe E.M., Filippini N., Griffanti L., Alfaro-Almagro F., Okell T., Sheerin F., Xie C., Mahmod M. (2021). Medium-term effects of SARS-CoV-2 infection on multiple vital organs, exercise capacity, cognition, quality of life and mental health, post-hospital discharge. EClinicalMedicine.

[40] Li T., Zhang Y., Gong C., Wang J., Liu B., Shi L., Duan J. (2020). Prevalence of malnutrition and analysis of related factors in elderly patients with COVID-19 in Wuhan, China. Eur. J. Clin. Nutr..

[41] Reiss J., Iglseder B., Kreutzer M., Weilbuchner I., Treschnitzer W., Kässmann H., Pirich C., Reiter R. (2016). Case finding for sarcopenia in geriatric inpatients: Performance of bioimpedance analysis in comparison to dual X-ray absorptiometry. BMC Geriatr..

[42] Tosato M., Marzetti E., Cesari M., Savera G., Miller R.R., Bernabei R., Landi F., Calvani R. (2017). Measurement of muscle mass in sarcopenia: From imaging to biochemical markers. Aging Clin. Exp. Res..

[43] He Y.C., Chen F. (2020). Rhabdomyolysis as Potential Late Complication Associated with COVID-19. Emerg. Infect. Dis..

[44] Li J.-W., Han T.-W., Woodward M., Anderson C.S., Zhou H., Chen Y.-D., Neal B. (2020). The impact of 2019 novel coronavirus on heart injury: A Systematic review and Meta-analysis. Prog. Cardiovasc. Dis..

[45] Bansal A., Kumar A., Patel D., Puri R., Kalra A., Kapadia S.R., Reed G.W. (2020). Meta-analysis Comparing Outcomes in Patients with and Without Cardiac Injury and Coronavirus Disease 2019 (COVID 19). Am. J. Cardiol..

[46] Wu T., Zuo Z., Kang S., Jiang L., Luo X., Xia Z., Liu J., Xiao X., Ye M., Deng M. (2020). Multi-organ Dysfunction in Patients with COVID-19: A Systematic Review and Meta-analysis. Aging Dis..

[47] Lau H.M., Lee E.W., Wong C.N., Ng G.Y., Jones A.Y., Hui D.S. (2005). The impact of severe acute respiratory syndrome on the physical profile and quality of life. Arch. Phys. Med. Rehabil..

[48] Gkekas N.K., Anagnostis P., Paraschou V., Stamiris D., Dellis S., Kenanidis E., Potoupnis M., Tsiridis E., Goulis D.G. (2021). The effect of vitamin D plus protein supplementation on sarcopenia: A systematic review and meta-analysis of randomized controlled trials. Maturitas.

[49] Liao C.D., Chen H.C., Huang S.W., Liou T.H. (2019). The Role of Muscle Mass Gain Following Protein Supplementation Plus Exercise Therapy in Older Adults with Sarcopenia and Frailty Risks: A Systematic Review and Meta-Regression Analysis of Randomized Trials. Nutrients.

[50] Chapple L.-a.S., Fetterplace K., Asrani V., Burrell A., Cheng A.C., Collins P., Doola R.e., Ferrie S., Marshall A.P., Ridley E.J. (2020). Nutrition management for critically and acutely unwell hospitalised patients with coronavirus disease 2019 (COVID-19) in Australia and New Zealand. Nutr. Diet..

[51] Cawood A.L., Walters E.R., Smith T.R., Sipaul R.H., Stratton R.J. (2020). A Review of Nutrition Support Guidelines for Individuals with or Recovering from COVID-19 in the Community. Nutrients.

[52] Yao Y., Luo Z., Zhang X. (2020). In silico evaluation of marine fish proteins as nutritional supplements for COVID-19 patients. Food Funct..

[53] Cengiz M., Borku Uysal B., Ikitimur H., Ozcan E., Islamoğlu M.S., Aktepe E., Yavuzer H., Yavuzer S. (2020). Effect of oral l-Glutamine supplementation on Covid-19 treatment. Clin. Nutr. Exp..

[54] Burgess L.C., Venugopalan L., Badger J., Street T., Alon G., Jarvis J.C., Wainwright T.W., Everington T., Taylor P., Swain I.D. (2021). Effect of neuromuscular electrical stimulation on the recovery of people with COVID-19 admitted to the intensive care unit: A narrative review. J. Rehabil. Med..

[55] Liu K., Zhang W., Yang Y., Zhang J., Li Y., Chend Y. (2020). Respiratory rehabilitation in elderly patients with COVID-19: A randomized controlled study. Complement Ther. Clin. Pract..

[56] Chaabene H., Prieske O., Herz M., Moran J., Höhne J., Kliegl R., Ramirez-Campillo R., Behm D.G., Hortobágyi T., Granacher U. (2021). Home-based exercise programmes improve physical fitness of healthy older adults: A PRISMA-compliant systematic review and meta-analysis with relevance for COVID-19. Ageing Res. Rev..

